# Poultry husbandry, water, sanitation, and hygiene practices, and child anthropometry in rural Burkina Faso

**DOI:** 10.1111/mcn.12818

**Published:** 2019-04-29

**Authors:** Aulo Gelli, Derek Headey, Elodie Becquey, Rasmane Ganaba, Lieven Huybregts, Abdoulaye Pedehombga, Marco Santacroce, Hans Verhoef

**Affiliations:** ^1^ International Food Policy Research Institute (IFPRI) Washington District of Columbia; ^2^ AFRICSante Bobo‐Dioulasso Burkina Faso; ^3^ London School of Hygiene & Tropical Medicine (LSHTM) London UK; ^4^ MRC Unit The Gambia at LSHTM Banjul Gambia; ^5^ Division of Human Nutrition Wageningen University Wageningen The Netherlands; ^6^ Cell Biology and Immunology Group Wageningen University Wageningen The Netherlands

**Keywords:** hygiene, nutrition, poultry

## Abstract

Poultry production in low income countries provides households with nutrient‐rich meat and egg products, as well as cash income. However, traditional production systems present potential health and nutrition risks because poultry scavenging around household compounds may increase children's exposure to livestock‐related pathogens. Data from a cross‐sectional survey were analysed to examine associations between poultry, water, sanitation, and hygiene practices, and anthropometric indicators in children (6–59 months; *n* = 3,230) in Burkina Faso. Multilevel regression was used to account for the hierarchical nature of the data. The prevalence of stunting and wasting in children 6–24 months was 19% and 17%, respectively, compared with a prevalence of 26% and 6%, respectively, in children 25–60 months. Over 90% of households owned poultry, and chicken faeces were visible in 70% of compounds. Caregivers reported that 3% of children consumed eggs during a 24‐hr recall. The presence of poultry faeces was associated with poultry flock size, poultry‐husbandry and household hygiene practices. Having an improved water source and a child visibly clean was associated with higher height‐for‐age *z* scores (HAZ). The presence of chicken faeces was associated with lower weight‐for‐height *z* scores, and no associations were found with HAZ. Low levels of poultry flock size and poultry consumption in Burkina Faso suggest there is scope to expand production and improve diets in children, including increasing chicken and egg consumption. However, to minimize potential child health risks associated with expanding informal poultry production, research is required to understand the mechanisms through which cohabitation with poultry adversely affects child health and design interventions to minimize these risks.

**Key messages**

- Poultry production can provide low income households with nutrient‐rich meat and eggs, as well as income. However, traditional production systems present potential health and nutrition risks since poultry scavenging around household compounds may increase children's exposure to livestock‐related pathogens.
- In rural Burkina Faso, though over 90% of households owned poultry only 3% of children consumed eggs during a 24‐hr recall. Chicken faeces were visible in70% of compounds and the presence of chicken faeces was associated with lower WHZ and no associations were found with HAZ.
- Research is required to understand the mechanisms through which cohabitation with poultry adversely affects child health and design interventions to minimize these risks.

## 1 INTRODUCTION

Global estimates indicate that in 2018 approximately 149 million children under 5 years of age were stunted and 49 million were wasted (UNICEF/WHO/World Bank, 2019). Trends suggest that scaling up nutrition specific interventions will not meet global targets for improving child nutrition outcomes (Bhutta et al., 2013), and actions in other sectors are required to accelerate progress. Nutrition sensitive interventions, including integrated agriculture and nutrition interventions, have strong potential due to the multiple ways they can influence the underlying determinants of nutrition, including income, women's empowerment, and the affordability of nutritious foods (Black et al., 2013; Ruel & Alderman, 2013). Livestock interventions, though often designed with income objectives, are increasingly being framed with nutritional rationales because of the high‐quality protein and nutrient density of many animal sourced foods (Iannotti et al., 2017; Iannotti, Muehlhoff, & McMahon, 2013; Iannotti, Lutter, Bunn, & Stewart, 2014; Food and Agriculture Organization of the United Nations, 2013; Murphy & Allen, 2003; Neumann, Harris, & Rogers, 2002). From the nutrition perspective, however, the impact of livestock interventions on the health environment is also a growing concern because greater ownership of livestock could lead to higher levels of contamination of the homestead compound or water supply with animal faeces (Ngure et al., 2013; Ngure et al., 2014; Schriewer et al., 2015), as well as increase the risk of environmental enteric dysfunction (EED; George et al., 2015), and other infections that might ultimately be harmful to nutritional status (American Thoracic Society, 1998; Donnelly, Berrang‐Ford, Ross, & Michel, 2015). EED is a subclinical disorder of the small intestine involving changes in gut structure, function, and immune system activation (Keusch et al., 2014; Prendergast & Kelly, 2012) that may be responsible for growth deficits during early childhood (Kosek et al., 2014).

In principle, targeted hygienic and sanitation practices could provide a protective barrier between animal faeces and children. Water, sanitation, and hygiene (WASH) interventions have been emphasized for combating diarrhoea and other water‐borne diseases, soil‐transmitted helminths, and EED. However, evidence‐based WASH interventions have focused mainly on reducing exposure to human faeces through improving access to and use of toilets, improving access to clean water, and encouraging appropriate handwashing methods. Relatively few existing WASH programmes place a strong emphasis on livestock management and animal faeces disposal, in part because scientists have hypothesized that human faeces are more important reservoirs for the pathogenic bacteria that most commonly cause diarrhoea (Curtis, Cairncross, & Yonli, 2000). However, a meta‐analysis of diarrhoea risk factors showed that exposure to livestock is an important risk factor for child diarrhoea (Zambrano, Levy, Menezes, & Freeman, 2014), and several observational studies link exposure to animals or animal faeces to child stunting or EED (George et al., 2015; Headey & Hirvonen, 2016; Headey et al., 2017). Moreover, some researchers have hypothesized that more‐widespread livestock faeces may be an important risk factor for an EED‐stunting pathway (Mbuya & Humphrey, 2016). Poultry are a particular concern given that scavenging poultry production systems, involving poultry roaming in and around the main household dwelling, expose young children to ingestion of chicken faecal matter or contaminated soil (Ngure et al., 2013). Evidence on these livestock‐related risks is however limited to the few observational studies cited above.

In light of the limited evidence base on the linkages between poultry rearing, WASH, and child nutrition, this study aimed to examine associations between these factors using rich survey data from rural Burkina Faso. The “Soutenir l'Exploitation Familiale pour Lancer l'Élevage des Volailles et Valoriser l'Économie Rurale” (SELEVER) cluster randomized control trial (cRCT) is an ongoing 5‐year study designed to evaluate the impact of an integrated agriculture–nutrition package of interventions (including poultry value chain development, women's empowerment activities, and a behaviour change communications strategy to promote improved diets and feeding, care, and hygiene practices) on the diets, health, and nutritional status of women and children in Burkina Faso (Gelli, Becquey, et al., 2017). Unlike standard household surveys, the SELEVER baseline survey includes a rich set of indicators around poultry husbandry and livestock‐related WASH factors, as well as child anthropometry and relevant determinants of child nutrition.

We use this extensive dataset to examine the following research questions: (a) How common is household poultry production and poultry consumption in young children in rural Burkina Faso? (b) How common is children's exposure to poultry faeces and what factors are associated with the presence of poultry faeces in shared spaces between poultry and young children? and (c) Are WASH characteristics and/or the presence of poultry faeces associated with anthropometric indices in young children? In addition to providing new evidence on the potential for livestock interventions to improve nutrition, the findings of this paper will also inform the analysis of the biomedical data from the SELEVER trial on the health risks associated with poultry husbandry that will be published separately.

## 2 METHODS

### 2.1 Country context

Burkina Faso is a low‐income country situated in Sahelian West Africa. The latest national nutritional surveillance survey found rates of stunting and wasting in children <5 years of 21% of 9%, respectively, and particularly poor infant and young child feeding practices (Direction de la nutrition/Ministere de la sante Burkina Faso(DNMS), 2016). Based on a cross‐country analysis of Demographic and Health Surveys (DHS) data, Burkina Faso was also found to have the second lowest dietary diversity score (the count of seven food groups) in the world for children under 24 months (Gelli, Headey, et al., 2017). In the 2010 Burkina Faso DHS, 80% of households owned poultry, yet only 14% of children 6–24 months had consumed poultry flesh and just 3% had consumed eggs in the past 24 hr (DHS, 2010).

### 2.2 Data sources

**Figure 1:**
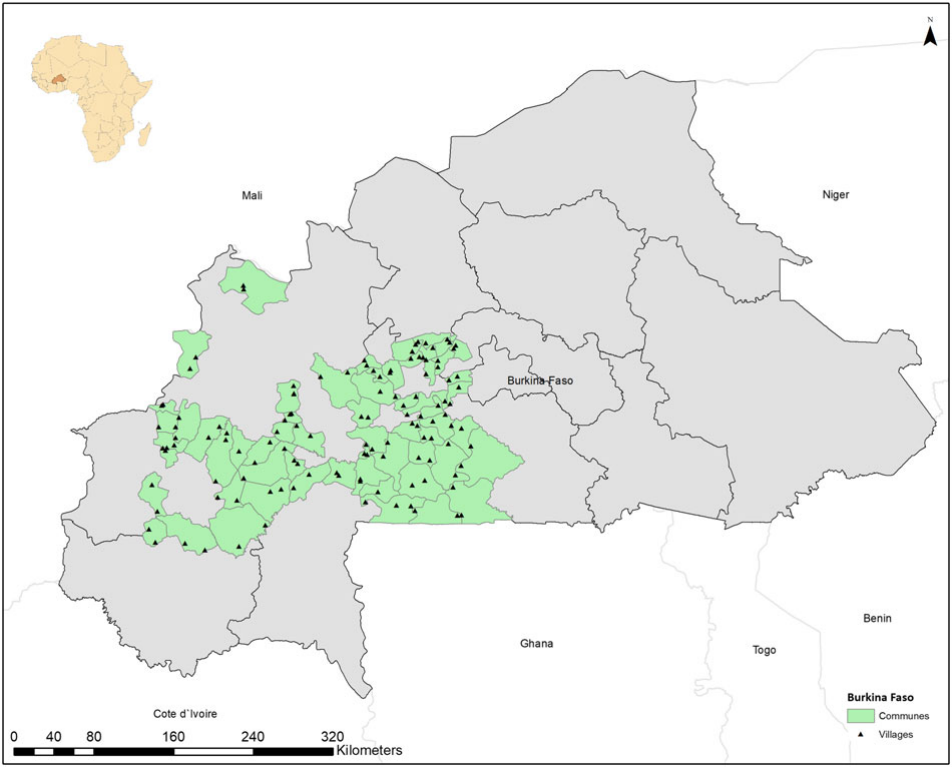
Map of the SELEVER study area and villages included in the study. Source: SELEVER trial

A baseline household‐ and child‐level survey for the SELEVER cRCT was undertaken in March 2017 in 120 rural communities across three regions of Burkina Faso including Boucle de Mouhoun, Centre‐Ouest, and Hauts‐Bassins. These regions were prioritized by the SELEVER implementers on the basis of the poultry production sector's potential to meet the demand for poultry in urban markets through greater commercialization (Some, 2015). The SELEVER trial protocol has been described in detail elsewhere (Gelli, Becquey, et al., 2017). Briefly, during the preparation stages of the SELEVER cRCT, 60 communes were selected from a pool of 79 communes that were available for scale‐up of the SELEVER intervention in the three targeted regions (Figure 1).

The criteria for selection were that a commune (a) was not included in the SELEVER pilot communes; (b) was classified as rural or peri‐urban (as classified in the national census); (c) had year‐round accessibility by road; and (d) had proximity to the Boucle de Mouhoun and Centre Ouest regions to facilitate implementation logistics (for the Hauts‐Bassins region communes only). Two villages were randomly selected within each commune from a list of villages with population sizes large enough to provide at least 15 households with children in the 2‐ to 4‐year age group based on data from the official population census of 2006. The definition of “household” in the context of Burkina Faso is complicated by the fact that many families share the same living compound and polygamous families are common. We adopted the use of the “restricted household” definition involving family members who normally share their meals together, which reflects the Living Standard Measurement Survey experience in collecting data in developing countries. Prior to the survey, a household census was conducted to identify all restricted households, all the large poultry producing households (defined as owning a poultry flock of over 20 chickens/fowls) in the community, and households with women aged 15–35 years with children in the 2–4 age group (see Gelli, Becquey, et al., 2017 for full details and the study protocol). Fifteen restricted households were then randomly selected from the household census for the survey interviews. The survey included child‐, caregiver‐, household‐, and village‐level data collection. Alongside a comprehensive survey at household level undertaken using a structured questionnaire, anthropometric data collection included all women of childbearing age (15–35 years) and all children under 5 years.

#### 2.2.1 Child anthropometry

The anthropometry variables in this analysis included height‐for‐age (HAZ) and weight‐for‐height (WHZ) *z* scores in children under 5 years of age. HAZ reflects cumulative measure linear growth relative to age; where a child with HAZ less than −2 is defined as stunted. WHZ is considered an indicator of current nutritional and health status; where WHZ less than −2 relates to being wasted, or suffering from acute malnutrition. Child weight was measured to the nearest 100 g using an electronic scale (SECA 876, Germany). Recumbent length of children <2 years of age and standing height of children >2 years of age were measured to the nearest 0.1 cm using portable fixed base stadiometers or length boards (SECA 417, Germany). All measurements were taken in duplicate by an anthropometrist and an assistant. Maternal weight and height were recorded using scales (SECA 877) and stadiometers (SECA 217, Germany), respectively. All measurements were practiced before the survey through standardization exercises. From the standardization sessions, interobserver and intraobserver variation of measurement error was documented, and the necessary corrections to procedures were made prior to the beginning of the survey. HAZ and WHZ scores were calculated using the 2006 WHO growth standards (WHO, 2006).

#### 2.2.2 Children's dietary diversity and poultry consumption

Consumption of poultry and diet diversity in children aged 6–23 months was assessed using an open qualitative recall of all food consumed in the previous 24 hr, considering the seven recommended groups for this population (including egg consumption as a separate food group) (WHO, 2007), and used the same method for children 24–59 months of age, considering 10 food groups (Food and Agriculture Organization of the United Nations & FHI 360, 2016). Information on poultry and egg consumption was collected separately from other animal source foods.

#### 2.2.3 Household WASH environment, poultry production, and other covariates

The presence of latrines, water sources, treatment of drinking water, and hand‐washing facilities was observed directly during the household interview using questionnaires developed in previous studies (Headey et al., 2017). Exposure to poultry and human faeces; cleanliness of mother's and child's hands, face, and clothes; and cleanliness of kitchen, house, latrine, and compound were assessed through direct observations undertaken at the time of the household interviews by trained enumerators using a prespecified checklist. Cleanliness was defined as absence of visible dirt. Mothers and children were classified as clean if their hands, faces, and clothes were observed to be clean. Direct observations were also undertaken of potential livestock contamination of water sources, the presence of poultry faeces in the compound and in the vicinity of the main food preparation area, keeping of livestock in the compound, and daily removal of animal faeces from the compound. Other WASH‐related characteristics including existence and evidence of use of a hand washing station and functional latrine were also recorded through direct observation. Structured interviews were also conducted with the mothers/caregivers in each household regarding childcare and WASH practices, including waste management and disposal of infant faeces, based on questionnaires implemented in similar studies (Headey et al., 2017). Data on poultry and livestock ownership were collected separately for women, men, and jointly owned poultry, using self‐reports over a 6‐month recall period, and then aggregated to provide household level indicators of total stock. The household survey also included a range of structured questionnaire modules including demographics, expenditures, education, and economic activities that provided the basis for a range of covariates for the multivariable regression models.

#### 2.2.4 Data collection

The survey questionnaires were programmed for data collection on electronic tablets using the SurveyBe Computer‐Assisted Personal Interviewing software (SurveyBe, UK). Survey enumerators were trained over a 3‐week period starting in February 2017. Sixty‐five enumerators were divided and trained in two groups, including those specializing in the household interview (*n* = 40) and those specializing on the anthropometry measurements (*n* = 25).

### 2.3 Statistical analysis

Exploratory descriptive analysis was undertaken to examine background characteristics in the study population. Summary statistics were presented as means ± *SD* for continuous variables and percentages for categorical variables. Nonparametric smoothening techniques were used to graphically explore non‐linear trends in the anthropometry outcome variables, using the local polynomial smoother with 95% confidence intervals (using lpolyci command in Stata). We identified a priori set of household, caregiver, and child level variables to include in the regression models based on the literature and 2013 Lancet Series conceptual framework for maternal and child nutrition (Black et al., 2013), including indicators characterizing poultry husbandry practices and the household environment. Regression analysis was undertaken using multilevel models that considered explicitly the hierarchical nature of the data and conceptual framework, including region, commune, and village level random intercepts to account for clustering (Goldstein, 2003). Analysis of the continuous HAZ and WHZ variables was undertaken using linear regression models, pooling all children 6–59 months, and separately for children 6–24 and 25–59 months. The analysis of the binary variable on the presence of poultry faeces in the compound was analysed using logistic regression models. The regression models were further adjusted for household socio‐economic status using household expenditures and mother's education. Statistical analysis was undertaken using Stata 15.0. Statistical significance was set at 5%, and all tests were two sided.

### 2.4 Ethical approval and consent to participate

Ethical clearance was obtained from the International Food Policy Research Institute IRB in Washington, DC (approved 12/26/2016, ref: IRB00007490) and the Comite de Recherche en Sante MS/MRSI in Burkina Faso (N°2016‐12‐142). The study was registered on the ISRCTN registry (ISRCTN16686478). Informed consent was requested from the each of the household heads prior to the interviews using a standardized form. All the survey tools were written in French, and the enumerators spoke both French and the local language.

## 3 RESULTS

### 3.1 Household characteristics

A total of 1,798 households and 3,490 children under 5 years were included in the baseline survey of the SELEVER trial. Average household size was nine household members, and only 4% households were female headed (Table 1). Thirty‐one percent of household heads had attended school, though the literacy levels in both local language and French were low. Approximately 46% of households were polygamous. On average, households spent 53% of total expenditure on food. Based on aggregate expenditures, 80% of households were found to be living below the international level poverty line (USD 1.25$ per capita per day PPP).

**Table 1:** Household characteristics and child anthropometry indices by age group in Burkina Faso

Level	Indicator	Mean (or %)	*SD*
Household			
	Household size	8.64	4.68
	No. of children <3 years	1.05	0.86
	No. of children 3 ≤ 6 years	1.09	0.83
	No. of children 6 ≤ 14 years	2.46	2.04
	No. of adults 15 ≤ 65 years	3.87	2.32
	No. of elders >65 years	0.17	0.43
	Polygamous household	46%	
	Household head		
	Male	96%	
	Age, years	43.26	13.31
	Any schooling	31%	
	Local language literacy	11%	
	French literacy	22%	
	Dwelling		
	Concrete floor	54%	
	Iron roof	75%	
	Mud walls	75%	
	Housing units	3.27	2.11
	Owned by household	94%	
	Expenditures per capita per day (USD)		
	Total	0.28	0.22
	Food expenditure/total expenditure	53%	0.25
	Poverty prevalence^a^	80%	
	Own livestock		
	Poultry	91%	
	Goats	78%	
	Cattle	59%	
	Donkeys	51%	
	Pigs	33%	
	Observations	1,798	
Child			
6–24 months	WHZ	−0.97	1.08
	HAZ	−0.88	1.41
	Prev. of wasting	17%	
	Prev. of stunting	19%	
	Observations	698	
25–60 months	WHZ	−0.53	0.98
	HAZ	−1.35	1.19
	Prev. of wasting	6%	
	Prev. of stunting	26%	
	Observations	2,069	

*Note*. SELEVER impact evaluation baseline survey.

Abbreviations: HAZ, height‐for‐age *z* scores; WHZ, weight‐for‐height *z* scores.

a Poverty line used to calculate the prevalence was USD 1.25$ per capita per day at purchasing power parity (PPP), equivalent to CFA 205.83 in 2015 (https://data.worldbank.org/indicator/PA.NUS.PPP). 1USD = 616 CFA (Forex 2/2017).

### 3.2 Child anthropometry

In children 6–24 months, the prevalence of stunting and wasting were 19% and 17%, respectively (Table 1). In children 25–60 months, the prevalence of stunting and wasting were 26% and 6%, respectively.

**Figure 2:**
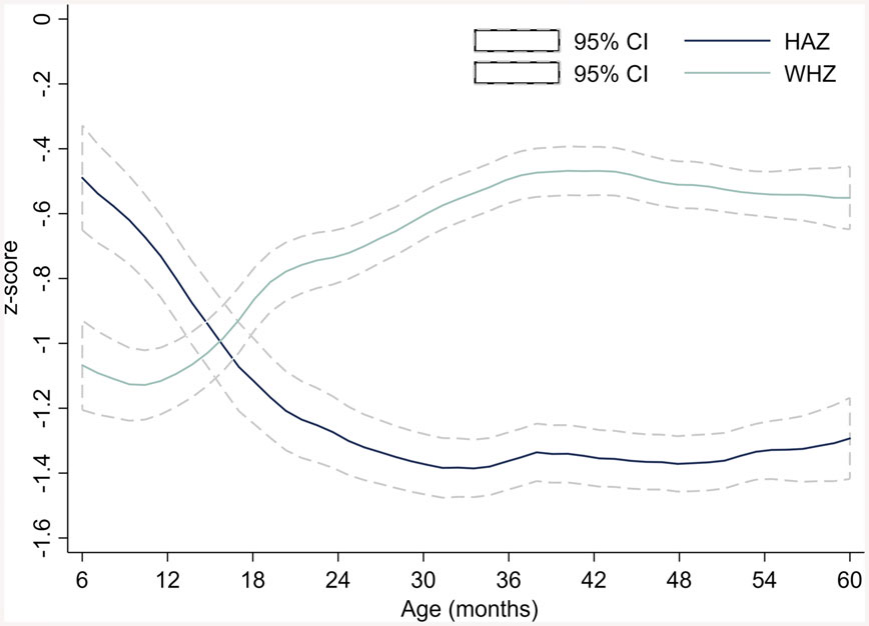
Estimated height‐for‐age z scores (HAZ) and weight‐for‐height z scores (WHZ) by age with 95% confidence intervals in Burkina Faso. Plots estimated using local polynomial smoother

Trends by age in HAZs and WHZs are shown in Figure 2. At 6 months, mean HAZ scores were approximately 0.5 *SD*s below the reference for this age group, whereas WHZ was nearly a full standard deviation below the reference for this age. HAZs then decline by age at a decreasing rate before stabilizing at approximately −1.4 *SD*s at 30 months. The WHZs profile followed a *U*‐shaped curve declining steadily until approximately 12 months of age (minima for WHZ −1.4 at 13 months) increasing between 12 and 40 months and subsequently stabilizing.

### 3.3 Poultry production and consumption

Poultry production was almost ubiquitous, with over 90% of households reporting poultry rearing in the study population (Table 1). However, median flock size was 25 chickens, with 25% of the population owning 10 or less chickens, and 75% of the population owning 46 or less chickens (Figure S1). On average, women owned far fewer poultry than men (Table S1), including fewer chickens (0.7 chickens, compared with 6.2, respectively), less chicks (0.9 compared with 8.8), and less young chickens (0.2 compared with 5.6). Average revenue from poultry for women was also considerably lower than that of males (106 CFA vs. 4,827 CFA for chickens, for example).

For children aged 2–4 years (the primary reference age group for the SELEVER trial), the mean dietary diversity based on 10 food groups was 3.98 (Table S2), and 35% of children over 24 months of age had a minimum dietary diversity (five or more food groups). All children had consumed a staple, 70% had consumed dark green leafy vegetables, and 36% had consumed a flesh food. Egg consumption in young children was extremely low, with only 3% of caregivers of children in the study population reporting their children consumed eggs in the day prior to the survey.

### 3.4 WASH environment

Fifty‐three percent of households reported having a borehole as their main water source, whereas 29% reported having an unprotected well (Table 2). Very few households (≤1%) reported treating their drinking water to make it safer. In 58% of households, respondents reported that livestock or wild animals had access to the area where the primary source of drinking water was located. Results from the household observations indicated that 41% of households owned a functioning pit latrine and 27% owned a clean pit latrine. Only 4% of households had a specific place where household members washed their hands, with only 1% of households having soap available for hand washing. Observations of mothers and children found that 72% of mothers had hands that were visibly clean at the time of the survey, compared with 37% of children. Caregivers reported that 58% of children had last defecated in the open. Only in 13% of households were children's stools disposed of in a latrine. Survey enumerators judged the compound to need sweeping at the time of the survey in 68% of households, and chicken faeces were visible in the compounds of 70% of households. Poultry were reported to be kept in the compound at some point during the day or night in 95% of households (Table S3) and were reported to be free to roam in the compound in 67% of households (a finding confirmed by the enumerator observations during the survey). Children and poultry were reported sharing the same spaces in 91% percent of households. Poultry were kept inside the household's living quarters (or actual home) in 9% of households. In 42% of households, the distance between where children slept and where poultry were kept at night was less than 10 m.

**Table 2:** Household water, sanitation, and hygiene environment and practices, Burkina Faso

Indicator	% of households
Sanitation	
Own a functioning toilette/latrine	41
Concrete latrine	20
Clean latrine (no faecal traces)	27
Latrine roof present and in good condition	10
Latrine walls present and in good condition	28
Latrine door present and in good condition	6
Latrine slab present and in good condition	28
Drinking water	
Main source is an improved, protected well	8
Main source is a sump/unprotected well	29
Main source is a borehole	53
Household does not treat drinking water to make it drinkable	90
Hand‐washing behaviours	
No specific place where household members wash their hands in compound	96
Traditional soap bar present where household members wash their hands	1
Other soap present	0
Hygiene	
Livestock have access to primary source of drinking water	58
Compound appears dirty	59
Garbage present in the home or compound	49
Perimeter of the house need sweeping and cleaning	68
Chicken faeces visible in the compound	70
Child or adult faeces visible in the compound	13
Soiled nappies visible in the compound	22
Dirty clothes visible in the compound	41
Outdoor food preparation area exists	62
If so, the soil around this area is dirty	40
Presence of chicken faeces in this area (radius of 3 m)	35
Animal faecal matter removed from compound daily	41
Garbage stored in a trash heap outside compound	40
Garbage disposed by throwing it into nature	21
Garbage disposed by burning trash pit	27
Observations	1,798

### 3.5 Factors associated with the presence of poultry faeces in shared spaces between poultry and young children

The presence of chicken faeces in the household compound was associated with not owning a latrine, having a child who is visibly dirty, the presence of dirty clothes and human faeces in the compound, livestock having access to the drinking water source, and the number of poultry kept in the compound (Table 3).

**Table 3:** Variables associated with the presence of poultry faeces in household compounds in Burkina Faso: Bivariate and multivariate multilevel logistic regression analyses

Indicator	Bivariate	*P*	Multivariate	*P*
Own latrine	0.52 (0.06)	<.001	0.63 (0.09)	.001
Main source of drinking water is a borehole	0.91 (0.11)	.473	0.97 (0.14)	.846
Dirty clothes visible in the compound	6.21 (0.96)	<.001	5.49 (0.93)	<.001
Human faecal matter visible in the compound	5.6 (1.39)	<.001	2.53 (0.71)	.001
Child open defecation	1.07 (0.12)	.520	1.13 (0.16)	.375
Child is fully clean	0.46 (0.05)	<.001	0.55 (0.08)	<.001
Livestock able to get into your primary source of drinking water	2.06 (0.26)	<.001	1.96 (0.27)	<.001
Keep poultry in compound	2.49 (0.31)	<.001	2.25 (0.39)	<.001
Log (no. of poultry)	1.25 (0.04)	<.001	1.17 (0.06)	.002
Log (no. of other livestock)	1.23 (0.05)	<.001	1.07 (0.06)	.246
Animal faecal matter removed from compound daily	0.51 (0.06)	<.001	0.75 (0.1)	.034
Log (per capita expenditure)	0.86 (0.07)	.065	0.87 (0.06)	.048
Mother completed primary education	0.65 (0.11)	.012	0.79 (0.16)	.233
Observations	1,734			

*Note*. Regression coefficients are presented as odds ratios, with standard errors in parentheses.

Abbreviation: Log, natural logarithm.

### 3.6 Associations between WASH, poultry/WASH characteristics, and child anthropometry

Tables 4 and 5 summarize the findings of the regression models for HAZ and WHZ scores. Having a borehole as the main source of water and having a visibly clean child were consistently associated with higher HAZ scores (though the association was not significant in the 6–24 months age group). No associations were found between HAZ, livestock ownership, and poultry hygiene related indicators. The presence of chicken faeces in the compound (including the food preparation area) was associated with lower WHZ scores (though the association was not significant in the 6–24 months age group). Socio‐economic status controls, as measured by the logarithm of household per capita expenditure and mother's completing primary education, were also positively associated with HAZ scores. In contrast, WHZ was only associated with household expenditures.

**Table 4:** Variables associated with height‐for‐age *z*‐score in children under 5 years, all and by age subgroup, in Burkina Faso (bivariate and multivariate multilevel linear regression analyses)

	All				6–24 months				25–60 months			
	Bivariate	*P*	Multivariate	*P*	Bivariate	*P*	Multivariate	*P*	Bivariate	*P*	Multivariate	*P*
Is a girl	0.12 (0.05)	.012	0.11 (0.05)	.021	0.27 (0.1)	.008	0.29 (0.1)	.004	0.05 (0.05)	.351	0.03 (0.05)	.541
Age	−0.01 (0.00)	<.001	−0.01 (0.00)	<.001	−0.05 (0.01)	<.001	−0.05 (0.01)	<.001	0.00 (0.00)	.525	0.00 (0.00)	.358
Borehole drinking water	0.15 (0.05)	.003	0.14 (0.05)	.004	0.18 (0.1)	.087	0.17 (0.1)	.098	0.17 (0.05)	.002	0.16 (0.05)	.004
Own latrine	−0.04 (0.05)	.444	−0.04 (0.05)	.383	−0.12 (0.11)	.245	−0.11 (0.11)	.313	0.02 (0.05)	.758	−0.01 (0.06)	.846
Child is fully clean	0.17 (0.05)	<.001	0.15 (0.05)	.002	0.17 (0.11)	.107	0.07 (0.11)	.502	0.18 (0.05)	<.001	0.18 (0.05)	.001
Keep poultry in compound	0.02 (0.07)	.779	0.02 (0.07)	.773	0.01 (0.15)	.932	−0.02 (0.15)	.906	0.00 (0.07)	.996	0.02 (0.07)	.793
Chicken faeces visible	−0.03 (0.06)	.568	0.00 (0.06)	.957	0.09 (0.12)	.458	0.06 (0.12)	.612	−0.08 (0.06)	.179	−0.03 (0.06)	.630
Animal faeces removed daily	0.02 (0.05)	.681	0.02 (0.05)	.670	−0.04 (0.11)	.736	0.01 (0.11)	.904	0.05 (0.05)	.362	0.03 (0.05)	.538
Log (no. of poultry)	0.03 (0.02)	.115	0.02 (0.02)	.237	0.05 (0.04)	.163	0.03 (0.04)	.463	0.01 (0.02)	.421	0.02 (0.02)	.427
Log (no. of other livestock)	0.04 (0.02)	.070	0.03 (0.02)	.102	0.07 (0.04)	.099	0.05 (0.04)	.273	0.02 (0.02)	.348	0.02 (0.02)	.381
Log (per capita expenditure)	0.06 (0.03)	.033	0.06 (0.03)	.028	0.04 (0.06)	.506	0.06 (0.06)	.345	0.07 (0.03)	.015	0.06 (0.03)	.032
Mother completed primary education	0.2 (0.08)	.010	0.22 (0.08)	.005	0.06 (0.18)	.731	0.16 (0.17)	.369	0.25 (0.08)	.002	0.25 (0.08)	.002
Observations			2,922				790				2,132	

*Note*. Standard errors in parentheses.

Abbreviations: HAZ, height‐for‐age *z*‐score; Log, natural logarithm.

**Table 5:** Variables associated with weight‐for‐height *z*‐score in children under 5 years, all and by age subgroup, in Burkina Faso (bivariate and multivariate multilevel linear regression analyses)

	All				6–24 months				25–60 months			
	Bivariate	*P*	Multivariate	*P*	Bivariate	*P*	Multivariate	*P*	Bivariate	*P*	Multivariate	*P*
Is a girl	0.07 (0.04)	.071	0.08 (0.04)	.041	0.32 (0.08)	<.001	0.32 (0.07)	<.001	−0.01 (0.04)	.904	0 (0.04)	.947
Age, years	0.01 (0.00)	<.001	0.01 (0.00)	<.001	0.02 (0.01)	<.001	0.02 (0.01)	.001	0.00 (0.00)	.050	0.00 (0.00)	.062
Borehole drinking water	0.07 (0.04)	.087	0.06 (0.04)	.125	0.14 (0.08)	.081	0.10 (0.08)	.214	0.04 (0.05)	.381	0.05 (0.05)	.317
Own latrine	0.05 (0.04)	.217	0.02 (0.04)	.609	−0.01 (0.08)	.876	−0.01 (0.08)	.891	0.06 (0.05)	.216	0.03 (0.05)	.477
Child is fully clean	−0.10 (0.04)	.013	−0.11 (0.04)	.003	−0.16 (0.08)	.049	−0.12 (0.08)	.134	−0.08 (0.04)	.057	−0.10 (0.04)	.018
Keep poultry in compound	0.04 (0.05)	.429	0.05 (0.06)	.356	−0.08 (0.11)	.482	−0.10 (0.12)	.385	0.09 (0.06)	.108	0.11 (0.06)	.069
Chicken faeces visible	−0.10 (0.04)	.026	−0.10 (0.05)	.022	−0.08 (0.09)	.362	−0.06 (0.09)	.483	−0.11 (0.05)	.031	−0.12 (0.05)	.025
Animal faeces removed daily	−0.02 (0.04)	.694	−0.02 (0.04)	.612	−0.09 (0.08)	.247	−0.10 (0.08)	.236	0.01 (0.04)	.882	0.01 (0.04)	.832
Log (no. of poultry)	−0.02 (0.01)	.151	−0.02 (0.01)	.123	−0.02 (0.03)	.522	−0.02 (0.03)	.563	−0.02 (0.02)	.252	−0.02 (0.02)	.179
Log (no. of other livestock)	0.01 (0.02)	.640	0.01 (0.02)	.456	0.04 (0.03)	.165	0.04 (0.03)	.259	0.00 (0.02)	.887	0 (0.02)	.833
Log (per capita expenditure)	0.1 (0.02)	<.001	0.09 (0.02)	<.001	0.15 (0.05)	.002	0.15 (0.05)	.002	0.08 (0.02)	<.001	0.08 (0.02)	.001
Mother completed primary education	−0.06 (0.06)	.296	−0.09 (0.06)	.139	−0.01 (0.13)	.934	−0.04 (0.13)	.735	−0.11 (0.07)	.110	−0.11 (0.07)	.100
Observations			2,922				784				2,137	

*Note*. Standard errors in parentheses.

Abbreviations: WHZ, weight‐for‐height *z* scores; Log, natural logarithm.

## 4 DISCUSSION

Despite the considerable potential of integrated agriculture and nutrition interventions to accelerate progress in addressing malnutrition, the prospects of improving diets of women and young children through interventions in livestock and poultry value chains have been largely ignored, and little attention has been paid in these interventions to improved poultry‐related hygiene promotion (Ruel, Quisumbing, & Balagamwala, 2018). Similarly, WASH interventions have traditionally focused on reducing exposure to human faeces, even though animal faeces are more widespread in typical village contexts (Gelli et al., 2017). This observational study was aimed at providing new evidence on the links between child anthropometry in young children, WASH, and poultry production practices in Burkina Faso.

### 4.1 Status of child nutrition, household WASH environment, poultry production, and consumption

Malnutrition was widespread in this sample of rural Burkinabe children, with stunting prevalence increasing with age in children under 24 months and stabilizing at around 26% in the 25–60 months age range, while wasting peaked among younger children (17% in 6–24 months) before declining to 6% in the 25‐ to 59‐month age range. The descriptive analysis also highlighted the high levels of poultry ownership at household level in this rural setting, consistent with the latest available DHS data from Burkina Faso. However, the new findings emphasized the relatively small flock sizes in the study population and the potential for increasing intensification, or efficiency of production. Moreover, despite the near ubiquity of poultry ownership, egg consumption in young children was practically nil (a finding also consistent with the latest DHS data). The low dietary diversity results also highlighted the monotonous staple‐based diets in children.

Although poultry are not yet an important direct contributor to children's diets, they may constitute a health risk. Chickens, and other small livestock, were found to roam freely throughout household compounds, mainly with the intent of finding enough feed to grow well and increase egg production, allowing households to save on purchased feed. Chicken faeces were visible in over 70% of households, suggesting that young children's exposure to animals and animal faeces is widespread and that children and poultry were often sharing the same spaces and interacted closely on a regular basis. These issues were also identified during structured observations of children in rural households undertaken during formative research in preparation for the SELEVER trial, including frequent exposures to poultry, poultry faeces, and soil (Gelli, Headey, et al., 2017). Chickens were also seen feeding both from plates that young children were eating from and from pots used to prepare meals. The primary strategy employed by women (generally the primary caregivers for both young children and chickens) to try to minimize the risk of children ingesting chicken faeces involved sweeping compounds. However, the frequency of sweeping the floor in compounds was found to vary considerably across households, with some households sweeping once or twice a day, and others less than once a month.

Overall hygiene levels in this study population were also very poor, including low rates of latrine ownership, adequate drinking water treatment, optimal handwashing, and waste management practices. Mothers' and children's hands were generally observed to be visibly dirty during the observation periods. These findings confirm the more qualitative results uncovered in formative research undertaken as part of the SELEVER study, where it was found that handwashing was rare for mothers, even after toileting and after disposal of animal faeces, and for children (Ngure et al., 2019). That research also found that children's faeces were also often ignored for long periods after defecation. Children's faeces were at times disposed of in latrines, though open defecation, disposal in garbage pits or tossing out in the compound was also very common. Children frequently mouthed their hands or objects that were visibly dirty, while sharing the compound with livestock and poultry, thus likely causing frequent exposure to a range of potential pathogens as they played on the dirt floor.

#### 4.1.1 Associations between the presence of chicken faeces and household, caregiver, and child characteristics

As expected, the presence of observable poultry faeces was strongly associated with poultry flock size and poultry‐husbandry practices, including rearing poultry in the household compound. The presence of chicken faeces also reflected general hygiene at household level, as highlighted by the negative associations between the presence of chicken faeces and general hygienic practices, including removing livestock faeces from the compound daily, preventing livestock access to the primary drinking water source, latrine ownership, and even a crude indicator on general child cleanliness, for example. These findings were consistent with evidence from similar studies in Bangladesh, Ethiopia, and Vietnam (Headey et al., 2017) and are suggestive of possible entry points for hygiene promotion interventions. Though socio‐economic status did appear to be associated with the presence of observable chicken faeces, surprisingly mother's education was not, perhaps a reflection of the very low levels of maternal education in the study population.

### 4.2 Associations between WASH environment and child anthropometry

The findings from the multiple regression models confirmed the important relationships between the WASH environment and child anthropometry indicators. Drawing water from a borehole, compared with having an unprotected well or other unsafe sources of water, was found to be associated with higher HAZ. There was also evidence that an indicator based on the direct observation of child cleanliness (including hands, face, and clothes) was positively associated with HAZ. Chicken faeces were found to be negatively associated with WHZ scores but not HAZ scores, suggesting a link between poultry faeces and child anthropometry indicators that has not been previously highlighted in the recent literature on this topic (Headey et al., 2017). This association was also found in children 25–60 months and not in children 6–24 months, and though this finding may be due to the smaller sample sizes in the 6‐ to 24‐month age group, it is suggestive of potential heterogeneities in mechanisms by age that will require further investigation. Despite the potential for livestock interventions to improve nutrition, no associations were found between livestock and poultry ownership levels and child anthropometry scores. The link between poultry faeces and WHZ is suggestive of a morbidity‐related relationship, highlighting a potential trade‐off of investments made to intensify poultry production. It will be important to unravel the mechanisms through which these relationships operate, as the impact pathways linking livestock and poultry production to child health and nutrition are complex, and also include uncertainties in the biological mechanisms involved (e.g., EED vs. diarrhoea). Moreover, these relationships may also affect other dimensions of child well‐being, including child development (Piper et al., 2017). The SELEVER trial includes a substudy designed to specifically examine the additional costs and benefits of adding an intensive poultry‐WASH related Behavior Change Communication (BCC) component alongside the standard integrated SELEVER package (Gelli et al., 2017). In particular, the intensive poultry‐WASH intervention will include two interlinked components, involving (a) a modified community‐led total sanitation approach (Kar & Chambers, 2008) incorporating poultry‐related messaging alongside the standard community‐led total sanitation approach; and (b) household level messaging on the importance of separating poultry and other livestock from young children and on protecting children from ingesting soil and animal faeces. Evidence from the trial will provide insights to optimize decisions involving flock size, sustainability, and cost‐effectiveness. Further analysis of the SELEVER baseline data is also ongoing, including biomedical data on infection biomarkers in children, which will provide important preliminary insights on the potential impact pathways and mediating factors involved.

One of the strengths of this study is the use of a wide range of non‐standard WASH indicators—particularly those related to livestock and poultry—which allows us to document the extent of livestock contamination of the homestead environment and to explore the potential relationships with WASH practices and children's anthropometry. The findings of this study are limited, however, by several important considerations. First, the study population was not representative for the Burkinabe population, although the descriptive data suggest that there were no important differences in terms of household characteristics with most recent country‐wide estimates. Second, due to the observational nature of this study, we are limited to describing associations between WASH‐related indicators and nutrition outcomes and interpreting these associations where they exist as indications of possible pathways, or modifiable factors, through which future interventions may operate. Third, though we provide some insights on the potential links between poultry husbandry and child anthropometry, including the potential risks associated with poultry faeces, the mechanisms through which exposure to poultry faeces may be harmful to children's growth and development are still unclear and are the subject of ongoing further investigation (Harper, Mutasa, Prendergast, Humphrey, & Manges, 2018). Fourth, the indicators of cleanliness of mothers, children, and the household environment from the direct observations in this study likely suffer from considerable measurement error because of their reliance on observations and judgements made by survey enumerators (Ruel & Arimond, 2002). The study attempted to reduce bias through extensive training, but further work is needed to validate these indicators as proxy measures of the health environment. Spot checks also have the limitation of measuring WASH conditions at a specific point in time, which may not be indicative of regular conditions. Indeed, a longer term “chronic” measure of livestock‐related WASH may be more relevant to explaining HAZ, and this fact may account for why we observed weak associations between the livestock‐related WASH indicators and HAZ. Finally, our analysis may have little external validity. Poultry ownership in our sample (and indeed in rural Burkina Faso as a whole) is extremely high, even by the standards of rural sub‐Saharan Africa. This may mean that most of this sample of children are highly exposed to livestock faeces, as well as generally poor WASH conditions, which limited the ability of these indicators to account for variation in nutrition outcomes.

## 5 CONCLUSIONS

Though informal poultry production is pervasive in Burkina Faso, the levels of both poultry production and consumption are low, suggesting that there is significant scope to further expand poultry‐based income generation for rural households and to improve diets in young children, including increasing chicken meat and egg consumption. Emerging evidence from this study also suggests that intensifying poultry production in contexts where children and poultry share living spaces may increase nutrition‐related risks for young children. In principle, more livestock‐oriented WASH programmes could provide an important barrier to prevent the transmission of pathogens from livestock to humans, although further research is required to explore these risks and the mechanisms involved.

## CONFLICTS OF INTEREST

The authors declare that they have no conflicts of interest.

## CONTRIBUTIONS

AG and DH led the study design; EB, RG, LH, AP, and HV provided inputs in the study design and manuscript preparation. MS supported the data analysis. All authors read and approved the final manuscript.

## Supporting information

Table S1: Livestock ownership by gender in Burkina Faso.
Table S2: Dietary diversity indicators, food consumed in 24 hrs prior to the survey, children 24‐48 m in Burkina Faso.
Table S3: Presence of livestock in the compound (*n* = 1,787), Burkina Faso.
Figure S1: Distribution of poultry flock size in the study population, Burkina Faso**.**
