# Supplementary material for: Poultry husbandry, water, sanitation, and hygiene practices, and child anthropometry in rural Burkina Faso
Source: Matern Child Nutr. 2019 Apr 29;15(4):e12818. doi: 10.1111/mcn.12818 (PMC6850613; doi:10.1111/mcn.12818)
Supplement: Supplementary file 1 — Table S1: Livestock ownership by gender in Burkina Faso. Table S2: Dietary diversity indicators, food consumed in 24 hrs prior to the survey, children 24‐48 m in Burkina Faso. Table S3: Presence of livestock in the compound (n = 1,787), Burkina Faso. Figure S1: Distribution of poultry flock size in the study population, Burkina Faso. [file MCN-15-e12818-s001.docx]

Supplementary table 1: Livestock ownership by gender in Burkina Faso.

|  |  | **Women** | | | |  | | **Men** | | | |
| --- | --- | --- | --- | --- | --- | --- | --- | --- | --- | --- | --- |
| **Poultry** | **Indicator** | **Mean** | **sd** | **n** |  | | **Mean** | | **sd** | **n** |  |
| Roosters | Stock amount (n) | 0.08 | 0.41 | 1,756 |  | | 2.05 | | 2.33 | 1,772 |  |
|  | Stock died/lost (n) | 0.02 | 0.24 | 1,770 |  | | 2.17 | | 6.1 | 1,798 |  |
|  | Stock Sold (n) | 0.05 | 0.34 | 1,768 |  | | 2.37 | | 4.76 | 1,795 |  |
|  | Revenue from stock (CFA) | 149 | 968 | 1,771 |  | | 8,116 | | 16,755 | 1,793 |  |
| Chickens | Stock amount (n) | 0.73 | 2.2 | 1,799 |  | | 6.17 | | 6.04 | 1,778 |  |
|  | Stock died/lost (n) | 0.07 | 0.44 | 1,757 |  | | 3.15 | | 8.25 | 1,797 |  |
|  | Stock Sold (n) | 0.05 | 0.33 | 1,766 |  | | 1.76 | | 5.3 | 1,798 |  |
|  | Revenue from stock (CFA) | 107 | 712 | 1,766 |  | | 4,827 | | 13,029 | 1,798 |  |
| Chicks | Stock amount (n) | 0.9 | 3.27 | 1,799 |  | | 8.40 | | 9.73 | 1,777 |  |
|  | Stock died/lost (n) | 0.36 | 1.99 | 1,786 |  | | 6.70 | | 12.3 | 1,784 |  |
|  | Stock Sold (n) | 0.00 | 0.00 | 1,797 |  | | 0.00 | | 0.05 | 1,792 |  |
|  | Revenue from stock (CFA) | 0.06 | 2.36 | 1,796 |  | | 20.0 | | 234 | 1,792 |  |
| Young chickens | Stock amount (n) | 0.15 | 0.80 | 1,751 |  | | 5.61 | | 8.33 | 1,798 |  |
|  | Stock died/lost (n) | 0.03 | 0.30 | 1,773 |  | | 2.43 | | 6.11 | 1,796 |  |
|  | Stock Sold (n) | 0.01 | 0.11 | 1,769 |  | | 1.48 | | 5.55 | 1,798 |  |
|  | Revenue from stock (CFA) | 25.6 | 311 | 1,777 |  | | 2,941 | | 11,332 | 1,797 |  |

Supplementary table 2: Dietary diversity indicators, food consumed in 24hrs prior to the survey, children 24-48m in Burkina Faso.

|  | **mean (or %.)** |
| --- | --- |
| Dietary diversity score | 3.98 (1.53) |
| Minimum dietary diversity (5+ food groups) | 35% |
| -consumed grains, roots, tubers in past 24 hrs | 99% |
| -consumed pulses (beans, peas and lentils) in past 24 hrs | 40% |
| -consumed nuts and seeds in past 24 hrs | 30% |
| -consumed dairy in past 24 hrs | 8% |
| -consumed flesh food in past 24 hrs | 36% |
| -consumed eggs in past 24 hrs | 2% |
| -consumed dark green leafy vegetables in past 24 hrs | 70% |
| -consumed vit-A rich f&v in past 24 hrs | 42% |
| -consumed other vegetables in past 24 hrs | 62% |
| -consumed other fruits in past 24 hrs | 8% |
| Observations | 1247 |

**Notes**: Where means are presented standard deviation is in parentheses.

Supplementary table 3: Presence of livestock in the compound (n=1,787), Burkina Faso.

| **Indicator** | **Dogs /cats** | **Poultry** | **Small livestock** | **Pigs** | **Cows** | **Donkey /horses** |
| --- | --- | --- | --- | --- | --- | --- |
| Present at any time during the day/night within compound | 64% | 95% | 88% | 40% | 65% | 63% |
| Animal kept inside compound | 49% | 88% | 64% | 16% | 30% | 31% |
| Animal free to roam within compound (self-reported) | 44% | 67% | 43% | 20% | 18% | 18% |
| Animal observed free roaming within compound | 32% | 66% | 32% | 12% | 8% | 9% |

**Supplemental Figure 1: Distribution of poultry flock size** **in the study population, Burkina Faso.**

**
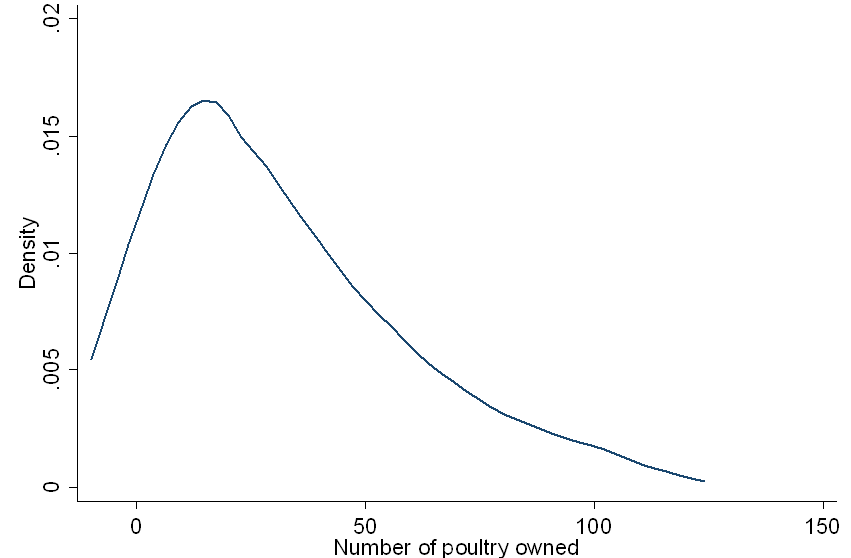
**

**Notes:** Univariate kernel density estimation, Epanechnikov kernel function with bandwidth = 10.
